# Effect of tACS on prefrontal neural activity is menstrual phase dependent in patients with premenstrual dysphoric disorder

**DOI:** 10.1016/j.brs.2022.07.052

**Published:** 2022-07-31

**Authors:** Justin Riddle, David R. Rubinow, Flavio Frohlich

**Affiliations:** Department of Psychiatry, University of North Carolina at Chapel Hill, Chapel Hill, NC, USA; Carolina Center for Neurostimulation, University of North Carolina at Chapel Hill, Chapel Hill, NC, USA; Department of Psychiatry, University of North Carolina at Chapel Hill, Chapel Hill, NC, USA; Department of Psychiatry, University of North Carolina at Chapel Hill, Chapel Hill, NC, USA; Carolina Center for Neurostimulation, University of North Carolina at Chapel Hill, Chapel Hill, NC, USA; Department of Neurology, University of North Carolina at Chapel Hill, Chapel Hill, NC, USA; Department of Cell Biology and Physiology, University of North Carolina at Chapel Hill, Chapel Hill, NC, USA; Department of Biomedical Engineering, University of North Carolina at Chapel Hill, Chapel Hill, NC, USA; Neuroscience Center, University of North Carolina at Chapel Hill, Chapel Hill, NC, USA

## Dear Editor,

**Fig. 1.**
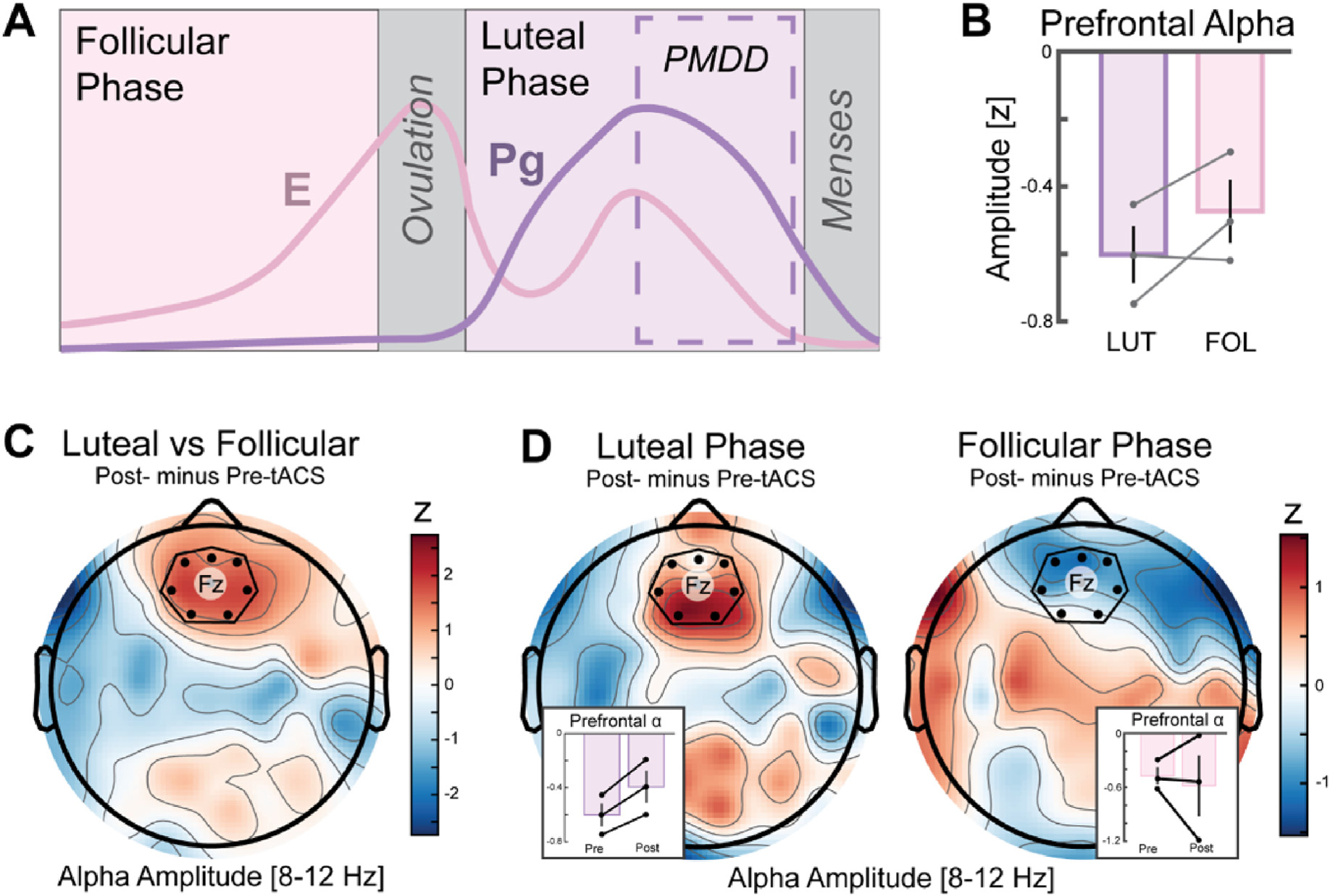
Case series in which tACS was administered to patients with premenstrual dysphoric disorder (PMDD). (A) PMDD is characterized by affective symptoms, depression, irritability, anxiety, and mood swings, in the late luteal phase of the menstrual cycle. E is estrogen and Pg is progesterone. (B) In our case series of three patients with PMDD, we found reduced prefrontal midline alpha amplitude in the luteal relative to follicular phase suggesting increased prefrontal control signaling concomitant with symptoms of PMDD. The y-axis is spatially normalized alpha amplitude via z-transformation. Dots represent each individual participant. (C) 40 minutes of bifrontal 10 Hz transcranial alternating current stimulation (alpha-tACS) resulted in a local modulation of alpha amplitude in the prefrontal midline as revealed by an interaction between stimulation time (post versus pre) and menstrual phase (luteal versus follicular phase). (D) Alpha-tACS increased prefrontal alpha amplitude in all three participants during the luteal phase, but showed high variance during the follicular phase. Prefrontal midline electrodes comprised Fz and its neighbors and are labeled by black dots and a heptagon.

In a recent clinical trial, we discovered that bilateral alpha-frequency (10 Hz) transcranial alternating current stimulation (alpha-tACS) decreased left frontal alpha oscillations only in patients within a major depressive episode and produced no significant change in euthymic control participants [1]. These findings suggest that the impact of tACS was dependent on the endogenous brain activity of the recipient, which is consistent with previous work modeling tACS as a weak perturbation that modulates the firing pattern of ongoing activity [2–4]. However, our previous study analyzed the difference in tACS effects between groups, patients within a major depressive episode versus euthymic control participants. It is unclear whether stimulation consistently modulates neural activity or if its impact is dependent on the affective state of the patient, e.g., when a patient is within a major depressive episode or outside of one. Premenstrual dysphoric disorder (PMDD) provides the opportunity to study the impact of stimulation on the brain activity of a single patient in two distinct affective states: while experiencing heightened depressive symptoms during the late luteal phase and while experiencing a reprieve from depressive symptoms during the follicular phase (Fig. 1A) [5–7].

Participants in this feasibility study were women with PMDD that were identified in a screening protocol for PMDD studies and recruited specifically to receive bifrontal alpha-tACS during the follicular and luteal phases of the menstrual cycle. To diagnose PMDD, we tracked affective symptoms, depression, irritability, anxiety, and mood swings, daily with the Daily Rating of Severity of Problems (DRSP) Form [8], an instrument that permits diagnosis of PMDD according to DSM-V criteria. PMDD was prospectively confirmed over 3 cycles to assure that at least five symptoms, one of which had to be a core affective symptom, met both severity threshold in the luteal phase and at least a 30% increase in symptom severity relative to the follicular phase, during which symptom ratings could not exceed mild. All participants were medication-free.

Investigations into the neural basis of PMDD have found that during the luteal phase the dorsal anterior cingulate cortex (dACC) shows elevated activity in anticipation of negative emotional stimuli [9,10]. Given that the amplitude of alpha oscillations is inversely related to cortical excitability [11], we expected that patients with PMDD may show decreased frontal-midline alpha amplitude during the late luteal phase corresponding with elevated activity in the medial prefrontal cortex and dACC. Critically, this divergence from equilibrium in the late luteal phase was the most likely activity pattern to be normalized by a weak perturbation, as recent evidence suggests that the impact of stimulation on brain activity is state-dependent [12–14] and stimulation may be particularly effective at destabilizing transient high symptom states by encouraging phase transition [15]. By applying alpha-tACS to bilateral prefrontal cortex, we hypothesized that stimulation would stabilize this divergence from equilibrium by increasing the amplitude of alpha-frequency oscillations over the frontal midline when applied during the late luteal phase, but not the follicular phase, of the menstrual cycle in patients with PMDD. In a case series reported here, three women with PMDD received 40 minutes of bilateral alpha-tACS during the follicular and luteal phase of their menstrual cycle (Pulvinar Neuro LLC, Chapel Hill, NC, USA). High-density electroencephalography during eyes-open and eyes-closed resting-state was collected before and after tACS (Electrical Geodesic Inc., Eugene, OR, USA). The study was single-blinded such that the experimenter was blind to the phase of the menstrual cycle of the participant, but the participant was not blinded as the stimulation was open-label and her menstrual phase was known to her.

Our analyses focused on the eyes-open resting-state recordings, as eyes-closed resting-state recordings are dominated by alpha oscillation generators in posterior cortex, which would dominate over anterior alpha generators [16]. Four minutes of EEG data before and four minutes after alpha-tACS were analyzed. Data were preprocessed similar to our previous reports [17]. The data were epoched into one-minute epochs, the Fourier transform was applied to each epoch, and the median spectral power density was calculated. Next, the average amplitude of the alpha frequency (8–12 Hz) was calculated for each electrode. Spatial normalization for each patient at each recording was applied across scalp electrodes using the z-transformation. Finally, activity over the frontal-midline was calculated as the average of the Fz electrode and the seven neighboring electrodes according to the standard 10–20 electrode system.

As a first step, we sought to identify the difference in neural activity that underlies the difference between the late luteal phase and follicular phase in women with PMDD. Thus, we analyzed the difference in prefrontal midline alpha amplitude prior to stimulation as a function of menstrual phase. We found that women with PMDD tended (two out of three; d = −0.77) to have decreased amplitude of alpha oscillations over the prefrontal in the late luteal phase relative to the follicular phase of their menstrual cycle (Fig. 1B). As alpha oscillations reflect neuronal inhibition [18], this finding is consistent with disinhibited, i.e., elevated, activity in the medial prefrontal cortex and dACC. Following bilateral stimulation with alpha-tACS, we found that prefrontal midline alpha amplitude was modulated as a function of menstrual phase (luteal versus follicular phase) and by the stimulation (post- versus pre-tACS) (d = 0.74) (Fig. 1C). When analyzing each phase of the menstrual cycle, alpha-tACS increased prefrontal midline alpha amplitude in all three women during the luteal phase (three out of three patients showed an increase, d = 1.87), but produced a variable response during the follicular phase (d = −0.35) (Fig. 1D). Thus, these findings are consistent with the state-dependent effect of tACS in that women with symptoms of PMDD showed the greatest impact of tACS on the targeted activity during the phase of the menstrual cycle that corresponded most acutely with their affective symptoms.

Altogether, this case series provides confirmatory evidence that the impact of tACS is context dependent and suggests that researchers or clinicians investigate the state of the brain as stimulation is administered. Critically, the analysis of brain activity as a function of menstrual phase prior to stimulation provides an understanding of the neural activity that was impacted by stimulation. In our previous study on major depressive disorder, we found that patients within a major depressive episode showed elevated left frontal alpha oscillations while processing positive valence images and that this pathologically elevated left frontal alpha activity was reduced by alpha-tACS [1]. By contrast, in this case series, patients with PMDD showed decreased alpha activity over the frontal-midline during the late luteal phase and this activity was increased by alpha-tACS. Based on these two different studies, alpha-frequency stimulation likely engages homeostatic processes that renormalize the distribution of alpha activity over the prefrontal cortex in a state-dependent manner [13,14,19]. Viewing the brain as a dynamical system, non-invasive brain stimulation may be particularly useful at destabilizing transient high symptom states by restructuring the energy landscape and encouraging phase transition [15].
